# Analysis of the Substrate Effect on the Zero-Backward Scattering Condition of a Cu_2_O Nanoparticle under Non-Normal Illumination

**DOI:** 10.3390/nano9040536

**Published:** 2019-04-03

**Authors:** Kaleem Ullah, Muhammad Habib, Lujun Huang, Braulio Garcia-Camara

**Affiliations:** 1 School of Electronic Science and Engineering, Nanjing University, Nanjing 210093, China; kaleem758@gmail.com; 2 Center for Micro and Nano Devices, Department of Physics, COMSATS University Islamabad, Park Road, Islamabad 44000, Pakistan; mhabiblhr@gmail.com; 3 Department of Material Science and Engineering, North Carolina State University, Raleigh, NC 27606, USA; Lujun.huang@adfa.edu.au; 4 Group of Displays and Photonic Applications (GDAF-UC3M), Carlos III University of Madrid, Leganes, 28911 Madrid, Spain

**Keywords:** nanoparticle, scattering, directionality, metamaterials

## Abstract

The presence of a substrate is one of the most important limitations of the real application of the directional conditions. These conditions allow the control of the spatial distribution of light scattering of nanoparticles. While the zero-forward condition is quite sensitive to any change of the surrounding medium, like the substrate, the zero-backward scattering seems to be less sensitive and very stable under normal illumination. In this letter, the zero-backward scattering condition was investigated on a homogenous Cu_2_O spherical subwavelength particle, both theoretically and experimentally. In particular, the influence of the substrate and the impinging direction on the angular distribution of light scattering under this directional condition were studied. We observed that the zero-backward scattering condition was also sensitive to the presence of a substrate beneath when a non-normal illumination was considered. We believe that our finding is quite interesting from a practical point of view and for the real implementation of directional scattering in various applications like cloaking, light-emitting devices, photovoltaic devices, bio-sensing, and many more.

## 1. Introduction

High-permittivity dielectric nanoparticles have been proposed as an alternative to the plasmonic ones because of their low absorption losses and magneto-electric response in the visible and infrared regions [1,2,3,4,5,6,7]. In addition, several multipolar components, i.e., dipolar, quadrupolar, and so on [8,9,10,11], and their interference are observed in the interaction of light with this kind of nanoparticles. The magnetic response of most natural materials is weak due to the negligible electronic spin at high frequencies resulting in small induced magnetic dipoles [12]. Consequently, the magnetic permeability of most natural materials in optics is assumed equal to that of vacuum. According to the Mie theory, high-refractive-index (i.e., Silicon, Germanium, etc.) sub-wavelength structures support strong magnetic resonances, despite their unitary relative permeability. The first experimental evidence in the visible region was given by using silicon nanospheres [13]; after that, this phenomenon has been studied with various materials and shapes of the particles [14,15,16,17,18,19].

An efficient control of the optical radiation at subwavelength scales, e.g., suppressing the unwanted backward scattering (BS) and enhancing the directional forward scattering (FS) is one of the most crucial issues and plays an important role for various applications, such as cloaking, bio-sensing, and superlenses. [20,21,22,23,24,25,26]. In the field of anomalous electromagnetic scattering, pioneering work can be traced to Kerker et al. [27], who systematically analyzed the light scattering of small magneto–dielectric spheres. The key ingredient for achieving zero-backward scattering in nanoparticles involves complete destructive interference between the electric and the magnetic dipolar responses to the incident wave which is known as the Kerker’s first condition [22,26,28,29,30]. The complexity of the practical observance of this phenomenon made it a theoretical utopia for several years. A few years ago, Kerker’s theory has been experimentally demonstrated [21,28,31]. However, its practical and realistic implementation are still a technological challenge. The presence of substrates [32] strongly influences the observance of the directional conditions. Zero-forward scattering rapidly vanishes as the substrate is included, so a real implementation of this condition requires ultra-low-refractive-index materials (e.g., aerogel) [32]. On the other hand, the zero-backward scattering seems to be quite stable to refractive index changes of the surrounding medium. However, most of the works use planar structures with a normal incidence direction [31]. This unequivocally restricts their application in real devices. For instance, the use of directional scattering in all-optical devices [33] requires large signal-to-noise ratios to optimize the operation. The substrate presence may limit it. Additionally, the illumination conditions of these devices depend on their integration in the system, so different angular illuminations may be observed. Therefore, further studies are required in this field.

In this work, we investigated the Kerker’s first condition on a Cu_2_O dielectric homogeneous sphere both experimentally and theoretically. In particular, we focused our study on the substrate effect and a non-normal incidence. Numerical simulations showed that this directional phenomenon becomes sensitive to the incidence angle when there is a substrate. Additionally, it also depends on the optical properties of the substrate. Experimentally, we also found an enhanced forward scattering and an almost null backscattering in a 184 nm-sized Cu_2_O sphere on a silicon dioxide (SiO_2_) substrate at the He–Ne laser wavelength of 632 nm with an incidence angle of 33°, satisfying our simulated results. We used apertureless scanning near-field microscopy (ASNOM) for obtaining the near-field scattering distribution of the Cu_2_O sphere. 

## 2. Theoretical Basis

The optical response of dielectric nanostructures is strongly influenced by the appearance and interaction of electric and magnetic multipolar resonances. About 34 years ago, Kerker et al [27] proposed that a complete suppression of the backscattered light from a spherical scatterer is possible under a certain condition. This requirement is known as the first Kerker’s condition or the zero-backward scattering condition. This establishes that the desired phenomenon arises at those incident wavelengths at which the electric and magnetic dipolar contributions match [31]. This result can be directly obtained from a simple analysis through Mie theory. When a linearly polarized light interacts with a spherical particle, the scattered intensity can be illustrated with respect to two polarized intensity elements in the scattering plane, as in Equations (1) and (2). The first scattering intensity (Equation (1)) is *I_TE_*, in which the electric field is perpendicular to the incident plane, whereas the second one (Equation (2)), *I_TM_*, corresponds to the electric field direction parallel to the incident plane. Assuming a dipole-like particle (particle radius, *R* << λ), the scattered field can be described by means of only the two first Mie coefficients, i.e., *a_1_* (electric dipole) and *b_1_* (magnetic dipole).

(1) $$I_{TE}=\frac{\lambda^{2}x^{6}}{4\pi r^{2}}{\left|\left(a_{1}+b_{1}\cos\theta \right)\right|}^{2}\sin^{2}\phi$$

(2) $$I_{TM}=\frac{\lambda^{2}x^{6}}{4\pi r^{2}}{\left|\left(a_{1}\cos\theta +b_{1}\right)\right|}^{2}\sin^{2}\phi$$

In these equations, *r* is the distance from the center of the particle to the observer, *a_1_* and *b_1_* are the first two Mie coefficients, *x* is the size parameter defined as *x = 2π·R/λ*, *θ* is the scattering angle, and *ϕ* is the angle between the incident electric field and the scattering plane [34]. For the backward scattering (*θ =* 180°), Equations (1) and (2) can be rewritten in the following form:

(3) $$I_{TE}(\theta =180{}^{\circ})=\frac{\lambda^{2}x^{6}}{4\pi r^{2}}{\left|\left(a_{1}-b_{1}\right)\right|}^{2}\sin^{2}\phi$$

(4) $$I_{TM}(\theta =180{}^{\circ})=\frac{\lambda^{2}x^{6}}{4\pi r^{2}}{\left|\left(-a_{1}+b_{1}\right)\right|}^{2}\sin^{2}\phi$$

It is very easy to understand from Equations (3) and (4) that when the dipolar moments match, the scattering intensity in the backward scattering direction will be zero for both incident polarizations. 

From a theoretical point of view, Kerker and co-workers considered free-standing particles without any substrate. Unfortunately, it can be seen that any modification of the refractive index contrast between the particle and the environment can prevent the observance of any directional effect. Several works have considered this effect, in particular in the minimum forward scattering [32,34]. 

## 3. Results and Discussion

**Figure 1:**
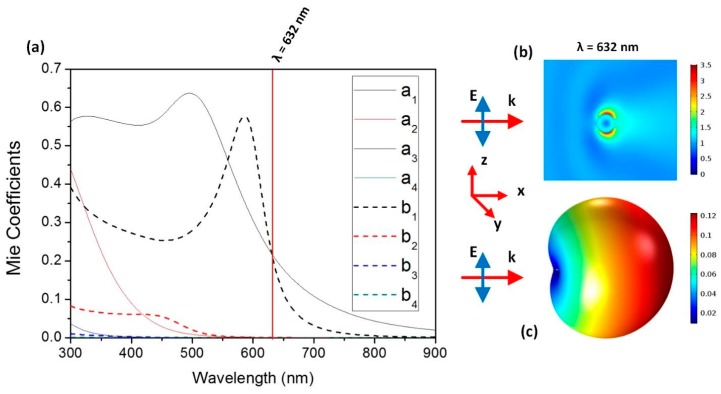
(**a**) Multipolar contributions towards the scattering cross section of a 184 nm-sized Cu_2_O sphere by applying x-polarized light at 632 nm wavelength. Calculated near-field (**b**) and 3D far-field (**c**) spatial distributions of the scattered field at a wavelength of 632 nm by FEM.

Figure 1a shows the first four multipolar contributions, both electric (*a_i_*) and magnetic (*b_i_*), to the scattering efficiency of a Cu_2_O particle with a spherical shape and a diameter of 184 nm, according to our experimental constraints. This calculation considers the complex refractive index of Cu_2_O in the considered spectral range, obtained from reference [35], while the surrounding medium is vacuum. Although the ratio between the particle size and the incident wavelength is not drastically low, it can be seen that multipolar coefficients with a higher order than the dipolar one [36] (*a_2_*, *a*_3_, … and *b_2_*, *b_3_*, *…)* give a negligible contribution to the scattering intensity for wavelengths larger than 400 nm. Interferences between the electric and magnetic dipolar modes, *a_1_* and *b_1_*, respectively, occur at the crossing points between both contributions. One of these crossing points appears at 632 nm, the He–Ne laser wavelength. At this wavelength, both the electric dipolar “*a_1_”* and the magnetic dipolar “*b_1_*” coefficients exhibit the same value, interfering destructively and causing a suppression of the back-scattering intensity. The satisfaction of the Kerker’s first scattering condition at this wavelength can also be checked either in the near- or in the far-field regime. By means of FEM (Finite Element Method) simulations, Figure 1b shows the near-field distribution around the considered particle at this wavelength, and Figure 1c shows the 3D far-field distribution. Both clearly show a zero-backward scattering. In particular, the near-field distribution (Figure 1b) shows two remarkable lobes at 90° and 270° with a minimum in the backward direction (180°).

**Figure 2:**
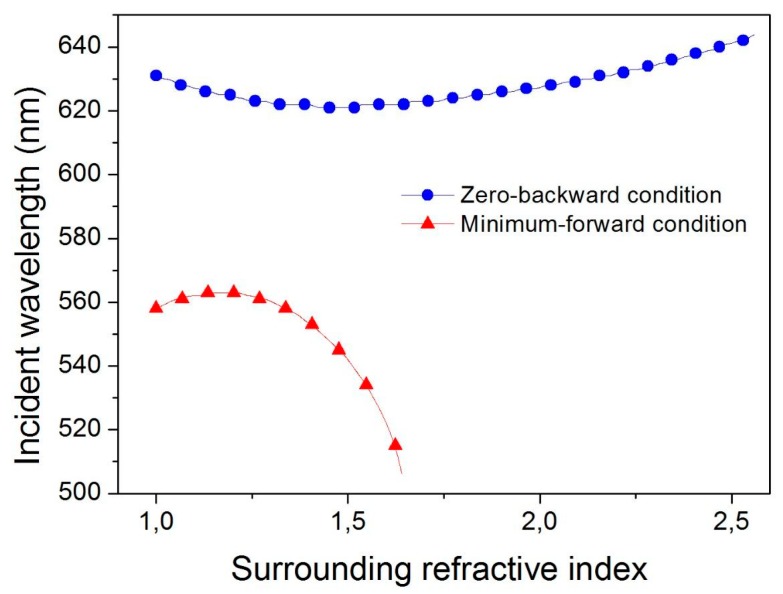
Comparison of the sensitivity of both directional conditions, the zero-backward and the minimum-forward scattering condition, with the external refractive index. The figure shows the incident wavelength at which any of the directional conditions are satisfied as a function of the refractive index of the surrounding medium. A Cu_2_O spherical particle with a diameter of 184 nm was considered in an ideal homogenous medium.

As the directionality conditions depend on the interference of the dipolar contributions, they strongly depend on the spectral position of them, as well as on the appearance of other multipolar contributions. This means that they are strongly dependent on the particle size and, particularly, on the refractive index contrast between the particle and the surrounding medium. In this sense and from a theoretical point of view, Figure 2 shows a comparison of the sensitivity of both Kerker’s conditions with the external refractive index. By applying Mie theory, we calculated the wavelengths at which Kerker’s conditions were satisfied as a function of the value of the surrounding medium, initially considered homogeneous. This figure clearly shows the strong dependence of the minimum-forward scattering condition on the refractive index, in such a way that it cannot be observed for values larger than 1.64. On the contrary, the zero-backward condition is much less sensitive to changes in the external refractive index. With a maximum sensitivity of 23.9 nm per refractive index unit (23.9 nm/RIU), it can be observed in a large range of the external refractive index. This makes this directional condition the most interesting one from a practical point of view. However, it is important to be careful about this slight shift of the Kerker’s condition wavelength, in particular, if a laser illumination is considered. 

**Figure 3:**
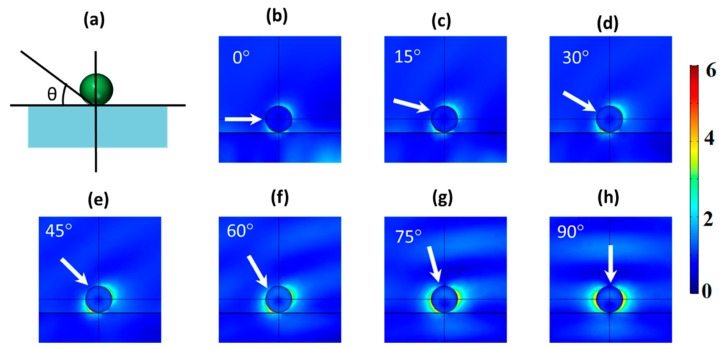
(**a**) A schematic showing how the angle of the incident beam varies with respect to the substrate. (**b**–**h**) FEM simulations of the scattered field of a Cu_2_O spherical particle with a diameter of 184 nm on a silicon dioxide (SiO_2_) substrate for different angles of the incident beam with respect to the substrate. The incident wavelength was 632 nm. White arrows label the incident direction.

**Figure 4:**
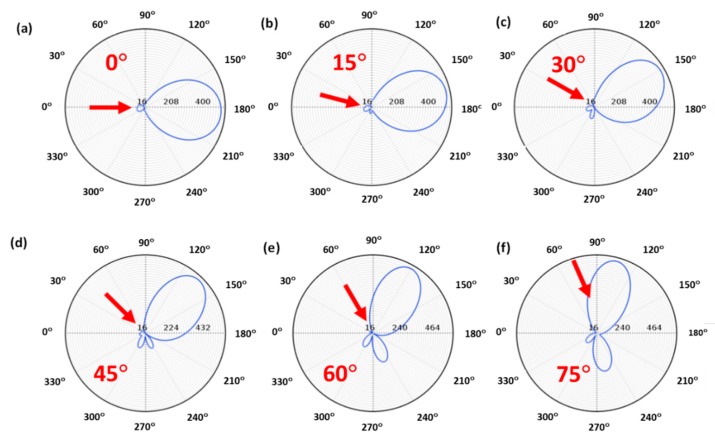
The far field polar plot of a 184 nm Cu_2_O particle, satisfying the zero-backward scattering condition, on a glass substrate and considering different incident angles with respect to the substrate: from 0° to 75° (**a**)–(**f**). The considered wavelength was 632 nm. Red arrows indicate the direction of the impinging light.

For a practical implementation in a wide range of applications, particles with directional scattering cannot be supportless, so the presence of a substrate is mandatory. The slight modification of the surrounding medium due to the inclusion of the substrate does not strongly affect the zero-backward scattering condition, as shown in Figure 2. In fact, it has been previously observed in other works [31]. However, from the best of our knowledge, there is no study about this dependence when the considered illumination is not normal to the substrate. In this sense, we explored, by using FEM simulations, the emergence of the first Kerker’s condition in the previously considered nanoparticle as a function of the incident angle (from 0° to 90°) and for two different substrates, i.e., silicon dioxide (Figure 3) and silicon (Figure 4). In both cases, the illumination wavelength was that of the He–Ne laser (632 nm) at which the directional effect was observed without a substrate (see Figure 1), and the thickness of the substrate was 1 µm. The low dependence of this condition on the substrate on normal incidence gave us confidence that this would still arise when we considered these substrates. This was corroborated as shown in Figure 3h and Figure 4d. Under normal illumination, a clear zero-backward scattering was observed in the near-field region of the nanoparticle. At other illumination angles, the behavior was more complex. 

Considering a glass substrate, the influence of the substrate under non-normal incidence was almost negligible, as shown in Figure 3. Particularly, as it can be seen in Figure 3b–e, the glass effects were negligible at low incident angles (<~45°), and zero-backward scattering was clearly observed in the near-field region. The two-lobe profile, observed in Figure 1b, was still observed in the presence of the substrate, although it was slightly modified by the presence of the substrate. On the other hand, for larger angles (Figure 3f,g), zero-backward scattering was not observed as perfect as before. Actually, under these angles, a minimum scattering was present close to the backward scattering, but it was not in this direction. This was remarkable at 60° (Figure 3f). As the incident beam was normal to the substrate (Figure 3h), zero-scattering appeared again in the backward direction, and the two-lobe profile, as in Figure 1b, appeared. This shows that destructive interferences between electric and magnetic modes were still produced, satisfying the Kerker’s condition. Regarding the scattering field of the considered scatterer in the far-field region, Figure 4 shows its spatial distribution at different angles of the impinging light. As in the near-field, it can be observed that the scattered intensity was strongly reduced in the backward direction at low angles (Figure 4a–d), with a maximum scattering in the forward direction. As the incident angle increased, the closeness of the dominant lobe, because of the substrate reflectivity, strongly hid this effect, as in Figure 4e, or eliminated it, as in Figure 4f. 

**Figure 5:**
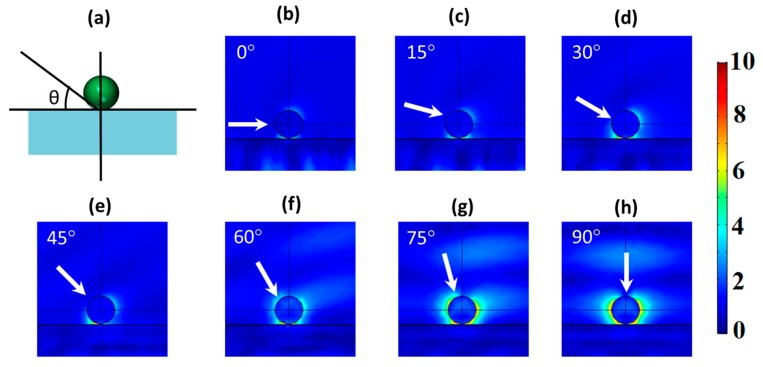
(**a**) Scheme of the geometrical conditions of the considered system. (**b**)–(**f**) FEM simulations of the scattered field of a Cu_2_O spherical particle with a diameter of 184 nm on a silicon substrate for different angles of the incident beam with respect to the substrate. The incident wavelength was 632 nm. White arrows label the incident direction.

**Figure 6:**
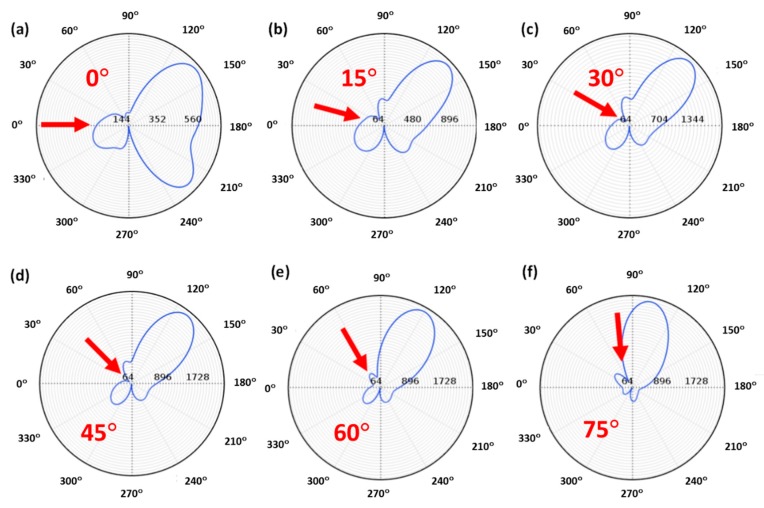
(**a**)–(**f**) Far field polar plots of a 184 nm Cu_2_O particle, satisfying the zero-backward scattering condition, on the silicon substrate for different incidence angles. The considered wavelength was 632 nm. Red arrows indicate the direction of the impinging light.

Considering substrates with larger values of their optical properties, the influence on the directionality was stronger than before, and the behavior was much more complex. This was the case of a silicon (*Si*) substrate (Figure 5). The smaller refractive index contrast between the particle and the substrate decreased the efficiency of arising resonant modes and the satisfaction of the directionality condition. In fact, a simple comparison between Figure 3 and Figure 5 shows that the typical two-lobe profile described in Figure 1b was not present in this second case until normal incidence was reached. In this sense, minimum backward scattering tended to appear at low angles (Figure 5b–d), while it disappeared for larger angles (Figure 5e–g), until reaching the normal incidence (Figure 5h) which resembled the directional condition. This complex behavior was also observed in the spatial distribution of the scattered field in the far-field. Figure 6 summarizes the main results of the far-field scattering considering the silicon substrate. For low incidence angles (0° to 45°), a dominant forward scattering was observed while backward scattering was reduced, as can be seen in Figure 6a–d. However, this was not as clear as in the case of the glass substrate. In contrast, for larger angles, i.e., 60° and 75°, there was minimum scattering as displayed in Figure 6e,f, but not in the exact backward direction.

**Figure 7:**
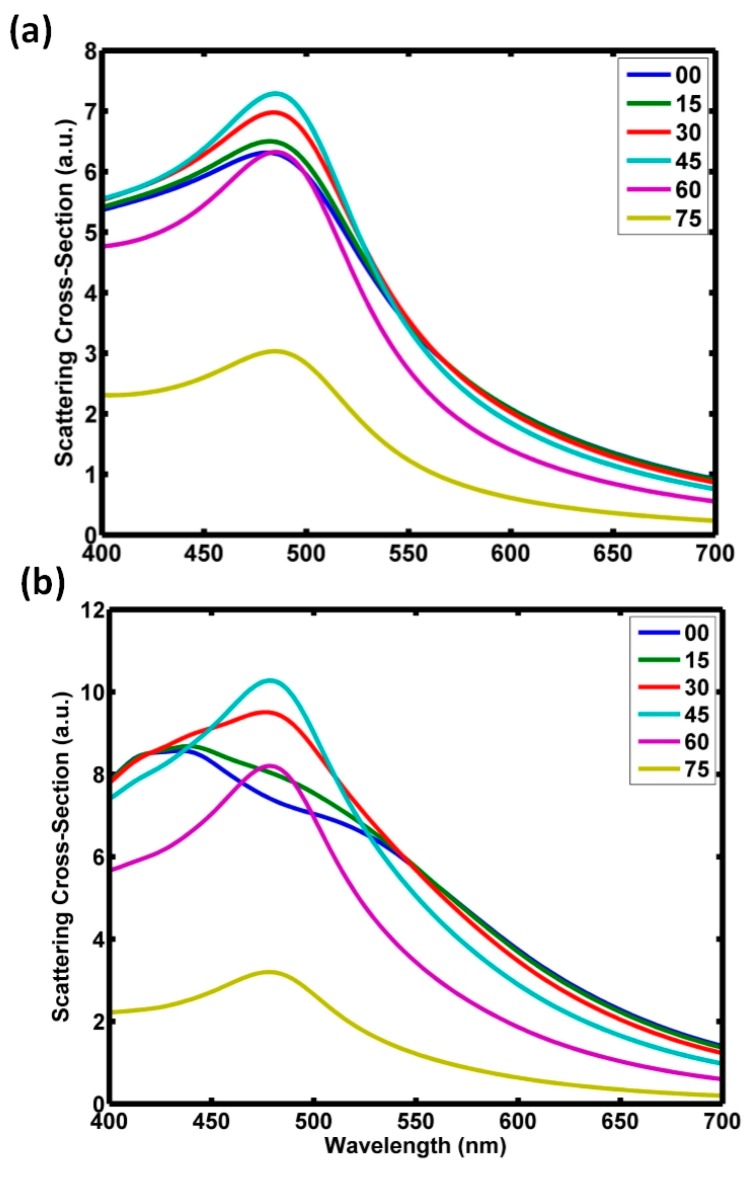
Scattering cross section (Q_SCS_) of a Cu_2_O particle on a (**a**) glass substrate or (**b**) silicon substrate.

**Figure 8:**
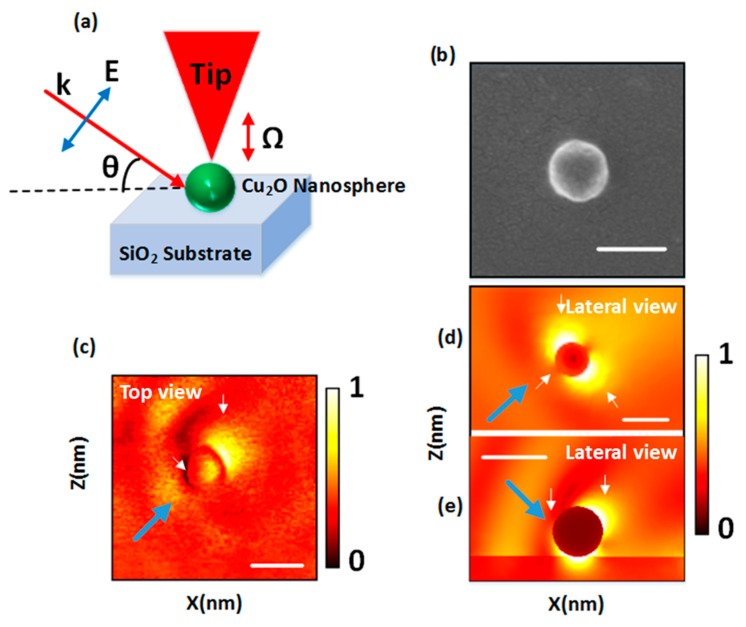
(**a**) A schematic of apertureless scanning near-field optical microscopy (ASNOM). A plane polarized light at the wavelength of 632 nm was used as incident field with an angle of incidence of 33°. (**b**) SEM image of the sample under test, (**c**) experimental image of the scattered amplitude delivered by ASNOM. The color bar is normalized with respect to the maximum value, and the length of the scale bar is 200 nm. This image shows a top view of the sample under test. (**d**,**e**) Simulated scattering amplitude without and with substrate, respectively, shown for comparison. These images are a lateral view of the sample on the XZ plane. Blue big arrows label the direction of the incident beam, while small white arrows highlight important features in the results.

To go deeply in this analysis, we also calculated the scattering cross section of a Cu_2_O particle on the glass and silicon substrate, as shown in Figure 7, for different incident angles. In the case of glass, a resonant peak appeared near 500 nm as can be observed in Figure 7a. As the incident angle changes, it seemed that the peak intensity changed, but its spectral position did not change. Then, we guess that, although the substrate did not affect the emergence of the dipolar modes and their coherent interaction, the modification of the spatial distribution of the scattered light by the substrate was responsible of the observed shift of the minimum scattering. In the case of the silicon substrate, the angular variation produced changes in the scattering cross section. This explains the important sensitivity of the zero-backward scattering on the incident angle, as observed before. In particular, when the incidence increased, the spectral structure became similar to the previous one, with a remarkable peak close to 500 nm, as can be observed in the Figure 7b. For small angles, the complexity of the spectra evolution of the scattering cross section was responsible for the non-dominant behaviors observed in both near and far fields (i.e., Figure 5b and Figure 6a), 

We also experimentally checked these results. In accordance with previous simulations, our experimental sample consisted of a Cu_2_O nanosphere, which was fabricated by a cost-effective method (see reference [37] for further information), on a SiO_2_ substrate (see Figure 8a). Copper (I) oxide (Cu_2_O) is a high refractive index material (*n*~3) with negligible losses in the visible and infrared region of the electromagnetic spectrum [38] and an interesting resonant scattering response [39]. Figure 5b displays a scanning electron microscopic (SEM) image of the sample showing that the particle was homogeneous and with a well-defined spherical shape. This method has been also used to estimate a particle size. In particular, this particle had a diameter of 184 nm. The scattering response of the sample was measured by means of ASNOM following the illumination scheme shown in Figure 8a. In ASNOM, a platinum probe, which is illuminated by a focused light beam, is used to scan the sample under study, collecting the scattered nea- field signal [40,41]. ASNOM images were obtained under the constant gap condition (constant vibration amplitude and constant average tip–sample distance). In this case, a linearly polarized light from a He–Ne laser (λ = 632.8 nm) was focused on the tip–sample junction with an incident angle of 33° with respect to the sample surface via an objective lens. This angle corresponds to a small angle at which we could still observe a zero-backward scattering, as can be seen in Figure 3. Back-scattered light from the tip–sample junction was collected by the same lens and homodyne-detected, giving separate intensity and phase information on the scattered field [42,43,44]. 

The scattering field amplitude obtained from ASNOM from a top view of the sample is shown in Figure 8c. The blue arrow labels the incident direction. A dominant forward scattering can be seen from the *Cu_2_O* sphere (see small white arrow in the right side of the particle). In addition, a reduction of light scattering in the backward direction can be intuited (white small arrow at left), although it is not clear due to the top view. FEM simulations without and with the substrate were carried out and are depicted in Figure 8d,e, respectively, for comparison. These images show a lateral view of the sample under test, where the big blue arrow shows the incident direction. As can be seen from Figure 8c, a strong scattering distribution in the forward direction of the sphere, as indicated by the small arrow on the right particle side, can be observed, while a suppression of the scattering in the backward direction can also be seen, indicated by the small arrow on the left side in Figure 8c. As a summary, while the isolated case (Figure 8d) presented two well-defined lobes in the near-field region (small white arrows just above and below the particle) and a dominant forward scattering as the distance increased, the presence of the substrate strongly disturbed the scattering distribution. However, we could still record a suppression of the scattering in the backward direction, as indicated by the small arrow in the left particle side in Figure 8e. A detailed comparison between experiment (Figure 8c) and simulations (Figure 8e) showed that the scattering distribution in the forward direction was much similar. Other differences in the scattering profiles between experiment and simulations were mainly because in the simulation, we sliced the particle from the center, whereas in ASNOM, the field distribution was measured on the surface of the particle.

## 4. Conclusions

In summary, we observed that the first Kerker’s condition, as well as the minimum-forward scattering condition, is affected by the presence of a substrate. However, in this case, the dependency arises when a non-normal illumination is considered. We numerically observed that, considering a substrate which is transparent to the impinging radiation, the directional scattering remains at low incidence angles, while at large incidence angles (except for the normal incidence), the lack of scattering is still there but it is slightly modified. This means that a destructive interaction is produced, but the substrate modifies its angular distribution. Considering a non-transparent substrate with a larger refractive index, the situation is more complex. In this case, the zero-backward condition is affected at low angular incidences, because the presence of the substrate modifies the spectral evolution of the scattering cross section and the emergence of multipolar modes. As the incident angle becomes larger, up to normal incidence, the directional scattering appears again. We checked our results with an experimental sample, showing that the directional scattering was still observed under a low angular incidence when a dielectric particle satisfying the Kerker’s condition was positioned on a glass substrate.

While a directional scattering of nanostructures is quite interesting in several fields, the presence of a supporting substrate has been one of the most important drawbacks of their real application. The minimum forward scattering has been almost completely discarded because of its strong dependency on the surrounding refractive index. On the contrary, the minimum-backward conditions have been observed as stable and promising. With this study, we suggest that a larger study should be done until a real implementation of the Kerker’s conditions in various applications. We trust that these directional conditions can offer novel and interesting possibilities in the future. We think that these results may be also applied to other interesting systems based on Kerker’s theory, like dielectric dimers [45] or core–shell nanoparticles [46].
